# A systems biology approach identified different regulatory networks targeted by KSHV miR-K12-11 in B cells and endothelial cells

**DOI:** 10.1186/1471-2164-15-668

**Published:** 2014-08-08

**Authors:** Yajie Yang, Isaac W Boss, Lauren M McIntyre, Rolf Renne

**Affiliations:** Department of Molecular Genetics and Microbiology, University of Florida, 2033 Mowry Road, Gainesville, FL 32610 USA

## Abstract

**Background:**

Kaposi’s sarcoma associated herpes virus (KSHV) is associated with tumors of endothelial and lymphoid origin. During latent infection, KSHV expresses miR-K12-11, an ortholog of the human tumor gene hsa-miR-155. Both gene products are microRNAs (miRNAs), which are important post-transcriptional regulators that contribute to tissue specific gene expression. Advances in target identification technologies and molecular interaction databases have allowed a systems biology approach to unravel the gene regulatory networks (GRNs) triggered by miR-K12-11 in endothelial and lymphoid cells. Understanding the tissue specific function of miR-K12-11 will help to elucidate underlying mechanisms of KSHV pathogenesis.

**Results:**

Ectopic expression of miR-K12-11 differentially affected gene expression in BJAB cells of lymphoid origin and TIVE cells of endothelial origin. Direct miRNA targeting accounted for a small fraction of the observed transcriptome changes: only 29 genes were identified as putative direct targets of miR-K12-11 in both cell types. However, a number of commonly affected biological pathways, such as carbohydrate metabolism and interferon response related signaling, were revealed by gene ontology analysis. Integration of transcriptome profiling, bioinformatic algorithms, and databases of protein-protein interactome from the ENCODE project identified different nodes of GRNs utilized by miR-K12-11 in a tissue-specific fashion. These effector genes, including cancer associated transcription factors and signaling proteins, amplified the regulatory potential of a single miRNA, from a small set of putative direct targets to a larger set of genes.

**Conclusions:**

This is the first comparative analysis of miRNA-K12-11’s effects in endothelial and B cells, from tissues infected with KSHV *in vivo*. MiR-K12-11 was able to broadly modulate gene expression in both cell types. Using a systems biology approach, we inferred that miR-K12-11 establishes its GRN by both repressing master TFs and influencing signaling pathways, to counter the host anti-viral response and to promote proliferation and survival of infected cells. The targeted GRNs are more reproducible and informative than target gene identification, and our approach can be applied to other regulatory factors of interest.

**Electronic supplementary material:**

The online version of this article (doi:10.1186/1471-2164-15-668) contains supplementary material, which is available to authorized users.

## Background

Kaposi’s sarcoma (KS) is an endothelial tumor and a major cause of AIDS patient death. Its associated herpes virus (KSHV, HHV-8) is a double strand DNA virus and a member of the γ subfamily of human herpes viruses [1]. KSHV can also infect lymphocytes, promoting transformation into primary effusion lymphoma (PEL) or Multicentric Castleman’s disease (MCD) in immunodeficient patients [2, 3]. The distinct pathological outcome of KSHV in two types of human tissues serves as a model system for studying cell type specific gene regulation.

In KS tumors and PELs, the majority of cells are latently infected and express viral genes only within a specific region of the viral genome: the KSHV latency-associated region (KLAR) [4–6]. This region encodes the latency-associated nuclear antigen (LANA, involved in latent DNA replication and episomal maintenance), v-Cyclin (cyclin D homolog that promotes S phase entry), v-Flip (promotes cell survival), the kaposin gene family (involved in cytokine mRNA stabilization and cell transformation), and 12 microRNAs (miRNAs). MiRNAs are small RNAs of 19–24 nucleotides that inhibit translation [7, 8] and induce degradation of mRNAs [9–11]. The genomic location of KSHV miRNAs and their abundant expression in KSHV-associated tumors suggests they play an important role in establishing latency and promoting KSHV pathogenesis.

**Figure 1:**
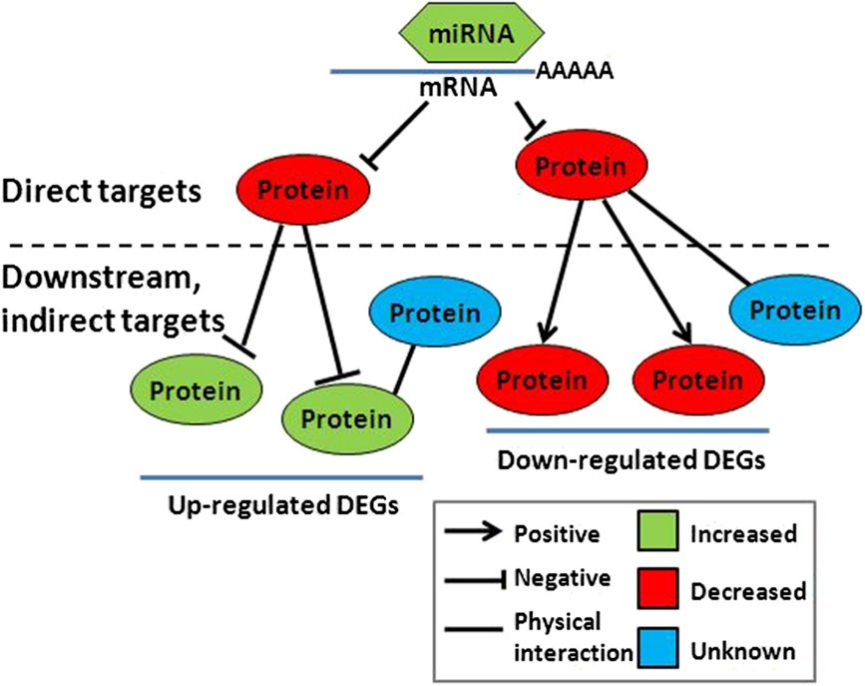
**MicroRNAs can affect GRNs directly and indirectly.** The regulatory effects of a miRNA are not limited to the direct RISC-dependent targeting. Both direct and indirect targets are integral components of GRNs and should be included in functional analysis. When a miRNA is over-expressed, its direct targets should be down-regulated. If the direct target is a repressor of downstream genes, then as a result of miRNA regulation, these genes will be de-repressed and their levels will go up (Upregulated differentially expressed genes or DEGs). On the other hand, genes downstream of activators and transcription factors will go down accordingly with the direct targets. In addition, proteins that physically associate with direct targets to function together in a complex may also be affected.

The first step in deciphering the functional role of a miRNA, is to identify its target genes. The 5′ sequence (especially bases 2–7, termed the “seed sequence”) of a miRNA, guides its complementary binding to the 3′UTRs of its target mRNAs and facilitates the repression of the latter in the RNA-induced silencing complex (RISC) [12–15]. Therefore, analysis of miRNA sequence properties can computationally predict its targets [16, 17]. Due to the short length of the seed sequence and the general disregard for tissue specific target-gene expression, bioinformatic approaches typically report large numbers of genes as putative targets of individual miRNAs reviewed by [18–20]. Greater than half of all protein coding genes in mammalian cells are estimated to contain multiple miRNA target sites [21]. Restricted by tissue specific gene expression, only a small fraction of putative targets are present in a specific cellular context (the *direct* targets) [22, 23]. The direct targets frequently do not function in isolation but interact with other molecules to form gene regulatory networks (GRNs). Accordingly, genes that are positioned at a lower level of the network hierarchy may also be functional targets even without the miRNA target site in their sequences (the indirect targets) (Figure 1).

This global regulatory effect can be captured by gene expression profiling after perturbing specific miRNA levels. The differentially expressed genes (DEG) reflect the global outcome of the miRNA regulation [13, 24]. *A priori* knowledge of molecular interactions is necessary to place the DEGs in the context for interpreting the joint effect of direct and indirect targets from a network perspective. A systems approach, which integrates secondary data with primary measurements of gene expression, can connect different layers of regulators from sparse and noisy expression profiles [25]. This approach is enabled by a variety of databases on DNA-protein and protein-protein interactions [26–28].

KSHV miR-K12-11 provides a unique model for studying tissue specific GRNs with regard to viral infection and pathogenesis. Its seed sequence is identical to cellular miR-155. Previous studies have identified similar functional targets of the two miRNAs [29, 30]. MiR-155 is a well-studied “oncomiR”, being associated with immune activation [31–33] and implicated in tumorigenesis [34–38]. MiR-K12-11 and miR-155 show mutually exclusive expression in KSHV infected tissues: miR-K12-11 is abundantly expressed in PEL cells, while miR-155 was detected in KSHV infected endothelial cells [30].

**Figure 2:**
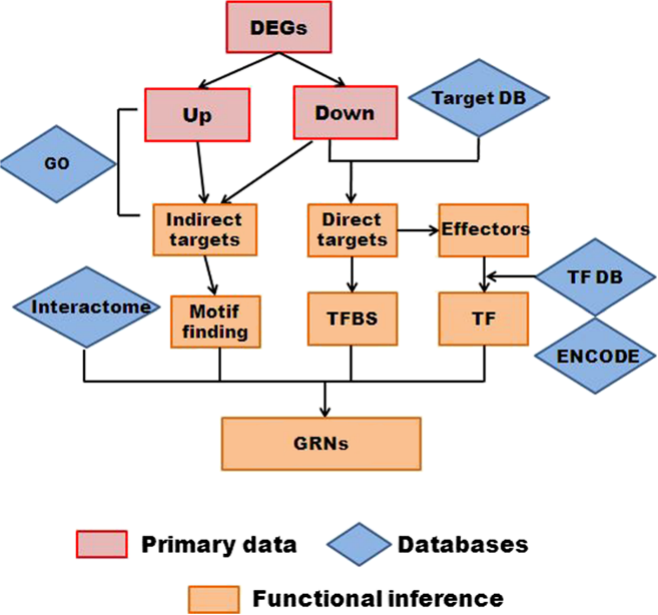
**Analysis pipeline.** By comparing the microarray profiles of miRNA-expressing cells and mock transduced cells, genes with significant changes were identified. The down-regulated genes with predicted miRNA binding sites were categorized as putative direct targets of miR-K12-11/miR-155. For direct targets that are known transcription factors, transcription factor binding sites (TFBS) were searched in the promoter regions of other affected genes. For those indirect targets, motif analysis within their sequences identified potential regulators. In addition, Gene Ontology and known protein-protein interactions help to build the gene regulatory networks (GRNs).

In this study, miR-K12-11 was expressed in KSHV negative human endothelial and B cells, close to physiological levels observed during KSHV infection. Tissue specific, as well as common target genes and pathways, were identified and the results were integrated with transcription networks, protein-protein interactome and signaling pathways. This systems approach (Figure 2) revealed that miR-K12-11 opposes host defenses and contributes to the proliferation and survival of KSHV infected cells by influencing key elements in cellular GRNs like TFs and signaling proteins. Our approach is applicable to a broader range of regulators of interest for understanding the GRNs in which they operate.

## Results and discussion

### Targetomes of miR-K12-11 in endothelial and B cells had little overlap in direct target genes, but shared many indirect targets in common pathways

**Figure 3:**
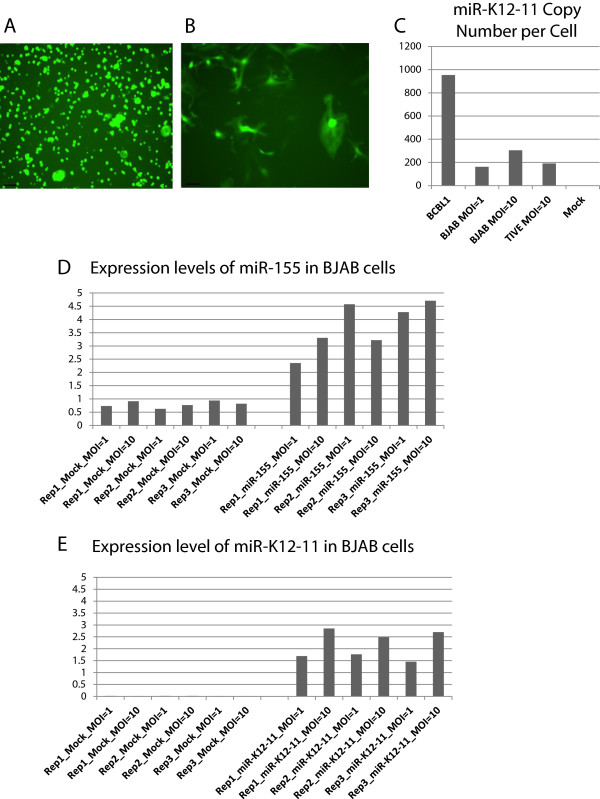
**Ectopic miR-K12-11 and miR-155 expression. A** and **B**. BJAB **(A)** and TIVE **(B)** cells stably express GFP after foamy virus transduction and purification by Fluorescence Activated Cell Sorter. **C**. Expression and copy number analysis of miR-K12-11 in transduced cells compared to the PEL cell line BCBL- using stem-loop qRT-PCR. The absolute numbers of miR-K12-11 from transduced cells was lower than in BCBL-1 indicating that ectopic expression was not to super-physiological levels. **D** and **E**. Expression levels of miR-155 in BJAB cells. There was endogenous expression of miR-155, although the ectopic miRNA expression was higher. Multiplicity of infection (MOI, i.e. copies per cell) did not result in consistent and significant changes in the miRNA expression levels, and was therefore not separately considered in further analysis. Y axis: relative quantity to the reference RNU66.

To mimic the cellular context of miR-K12-11, we moderately expressed miR-K12-11 in cells of lymphatic origin (BJAB) and endothelial origin (TIVE), using a recombinant retroviral vector with bi-cistronic miRNA and GFP genes. The constant detection of GFP in the transduced cells indicated stable expression of the miRNA gene (Figure 3A and 3B). Quantitative PCR results further confirmed the ectopic expression of miR-K12-11 in both BJAB and TIVE cells (Figure 3). Specifically, the retroviral transduction approach imitates miRNA expression under physiological conditions, unlike transfection experiments that excessively over-express the miRNA and trigger off-target effects [39–42]. In our experiment, the copy numbers of ectopic miR-K12-11 were lower than in BCBL-1 cells (KSHV infected B cell line isolated from cancer patients with PEL), indicating that it was not expressed at superphysiological levels (Figure 3C). To compare the GRNs of miR-K12-11 to those of miR-155, we also carried out retroviral transduction for miR-155. In BJAB cells, miR-155 was significantly expressed over the endogenous level. The miR-155 transduced TIVE cells, however, did not show significantly increased miR-155 levels over endogenous expression, preventing further analysis on miR-155 in this cell line.

In addition, the over-expression of miR-K12-11 did not affect the baseline expression of miR-155 in BJAB cells but was repressive in TIVE cells (Additional file 1: Table S4).

**Figure 4:**
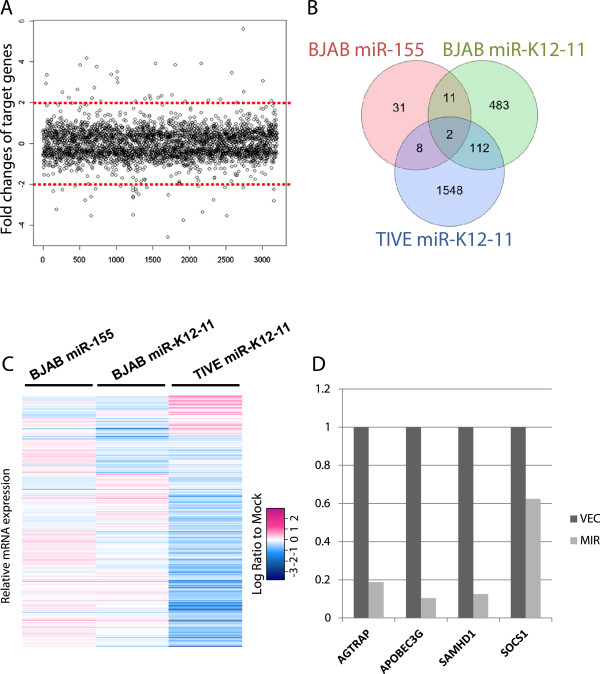
**Overall effect on the transcriptome after ectopic miRNA expression. A**. miRNA effects are quantitatively moderate. The fold change of expression levels for most DEGs was below 2-fold. **B**: Venn diagram showing common gene expression changes between cell lines. **C**. Heatmap showing the expression change compared to the mock samples for all down-regulated differentially expressed genes (DEGs) that are also predicted to be miR-155/miR-K12-11 targets. Most DEGs show strong tissue specificity. **D**. Verification of microarray measurements by qPCR on four previously reported miR-155/miR-K2-11 targets.

RNA samples for microarray analysis were collected from four biological replicates of BJAB cells expressing miR-K12-11or miR-155, TIVE cells expressing miR-K12-11, and corresponding mock controls. All samples were successfully hybridized and showed statistical agreement among biological replicates (Pearson correlation > 0.9, Spearman correlation >0.9, weighted kappa >0.7). Differentially expressed genes (DEGs) were determined using paired comparisons with FDR < 0.05 as the significance cutoff. Among the total 13,793 genes surveyed by the array, 141 were DEGs responsive to miR-155 in BJAB cells, and miR-K12-11 affected 1,215 and 3,189 genes in BJAB and TIVE cells, respectively (Table 1; Additional file 2: Table S1). Endogenous expression of miR-155 is expected to affect its target genes, and therefore few genes were expected to be differentially regulated by the addition of ectopic miR-155. This, and the target specificity beyond the seed sequence, led to few overlapping DEGs between miR-155 and miR-K12-11 in BJAB cells (Figure 4). The fold changes of the DEGs were mostly modest: 91% of the DEGs caused by miR-K12-11 had less than a 50% change at the RNA level in TIVE cells (Figure 4A). The effect was even more moderate in BJAB cells, with 97% of the DEGs changing less than 50%. The small fold changes were consistent with previous reports [7, 11] that miRNAs act as fine tuners of gene expression.

**Table 1:** **Number, direction and fold change (FC) of differentially expressed genes (DEGs)**

miRNA	Cell type	Direction	FDR <0.05	FDR <0.05 and FC > 1.2	FDR <0.05 and FC > 2
**miR-K12-11**	TIVE	Down	1607	1332	151
		Up	1582		
**miR-K12-11**	BJAB	Down	608	325	21
		Up	607		
**miR-155**	BJAB	Down	52	37	4
		Up	89		

DEGs were identified using a paired test with significance cutoff FDR < 0.05.

Genes commonly affected by miR-K12-11 between BJAB and TIVE were relatively few (<20%; Figure 4B and 4C). We also compared our DEGs with multiple miR-155/miR-K12-11 perturbation studies (Additional file 2: Table S1). A similar study expressing miR-K12-11 in BJAB transductants [29] had 40% of the DEGs (19 out of 48) shared by our miR-12-11 targets in BJAB. No such studies have yet been carried out in endothelial cells. In other cell types, few overlapping genes were identified, likely because the tissue specific transcriptomes are different (Evidence on tissue specific transcriptome profiles is abundant, e.g. in [43, 44]). These results demonstrated the tissue specificity of miRNA target genes and the importance of targetome identification in relevant cell types.

Direct targets of miRNAs are expected to be repressed through sequence complementarity. We identified these genes as down-regulated DEGs that also contained seed matches, as predicted by a union of bioinformatics algorithms (Additional file 2: Table S2). The repression of four such genes was verified by qPCR. They are AGTRAP (angiotensin), APOBEC3G (controls RNA processing), SAMHD1 (regulates TNF- α proinflammatory responses) and SOCS1 (cytokine suppressor) (Figure 4D). AGTRAP and SAMHD1 are validated targets of miR-K12-11 [29]. MiR-155 is able to suppress SOCS1, a suppressor of cytokine signaling [45] and AID, a member of the same family of deaminases with critical functions in adaptive and innate immunity as APOBEC3G [46–48].

Comparison between the computational target prediction and DEGs found only a small portion of the DEGs attributable to direct targeting. The number of up-regulated genes was about the same as the number down-regulated. Down-regulated genes and predicted targets were associated in TIVE cells (chi-square test p = 0.0128 for TIVE, p = 0.3227 for BJAB) (Additional file 1: Table S4). Several factors may contribute to the predicted but not observed targets: false predictions by the bioinformatics algorithms; true targets that are tissue specific, false negatives for the tests of differential expression; or targets subject to translational control not measured by mRNA profiling.

Despite the limited overlap between DEGs in TIVE and BJAB cells, miR-K12-11 targeted many common pathways in these two cell types (Additional file 2: Table S3). By comparing Gene Ontology (GO) terms with DEGs using Fisher’s exact test (significance cutoff P < 0.05; GEO accession: GSE59412)”, we found carbohydrate metabolism among the top enriched pathways in both cell types (Additional file 2: Table S3). Delgado et al. [49] reported that KSHV latent infection of endothelial cells strongly induced the Warburg effect, the phenomenon that cancer cells increased glycolysis to meet their energy needs [50, 51]. Glycolysis was also identified as the top enriched biological process in a comprehensive miRNA targetome analysis in KSHV infected PEL cells [52]. Taken together, this evidence suggests that miR-K12-11 is an important regulator for the metabolic change after KSHV infection in both endothelial and B cells.

### Effect of miR-K12-11 was amplified by transcription factors and protein interactions

GO enrichment analysis identified sequence-specific transcription factors (TFs) and protein binding among the top molecular functions of direct miR-K12-11 targets in both BJAB and TIVE cells (Fisher’s exact test p < 0.05), leading us to hypothesize that the indirect targets were produced by transcriptional regulation and protein interactions. Enrichment of TFs in miRNA targets have been reported for plants [53], insects [54] and human [55]. MiRNA regulation can control TF levels [56–59] and explains the importance of the 3′UTR for the stability of TFs [60, 61]. By binding to promoter elements and interacting with cofactors, TFs regulate the expression of a large number of genes and are able to amplify the effect of the initial miRNA targeting event. While miRNA regulation can result in an indirect effect of both up-regulation and down-regulation (Figure 1), negative regulators of gene expression are more context-dependent and difficult to prove. Here we focused on the feed-forward GRNs in which the components consistently change towards the same direction.

In TIVE cells, we identified multiple cancer associated TFs that were down-regulated and thereby amplified the regulatory effects of miR-K12-11. We identified CEBPβ, E2F1, PAX6, RELA (also known as NF-κB p65), and STAT1 using a combination of DEGs and target prediction. CEBPβ is a previously confirmed target for both miR-155 and miR-K12-11 in B cells and in the context of human hematopoiesis [62, 63]. E2F1 is a master regulator of cell cycle. PAX6 is involved in tissue specification during early development. RELA promotes DNA repair and resistance to apoptosis through the regulation of anti-apoptotic proteins. STAT1 is required for antiproliferative activity, immune surveillance and tumor suppression. Repression of these key regulators involved in cancer by miR-K12-11 may help the establishment of latency and play a role in KS tumorigenesis. Moderate down-regulation of these five TFs by miR-K12-11 should result in decreased expression of their downstream genes.

**Figure 5:**
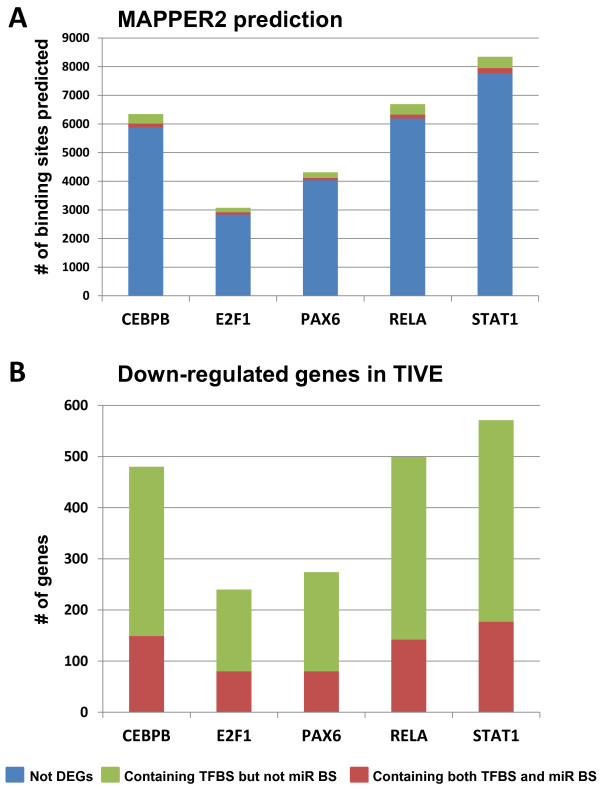
**Transcription factor binding sites (TFBS) prediction for TFs directly targeted by miR-K12-11 in TIVE cells.** MAPPER2 predicted thousands of genes with binding sites for each of the five TFs. **A**. All predicted sites with the genes not on the array, not expressed, or were not differentially expressed between miRK12-11 induction and control, or located in inactive chromatin regions according to ENCODE data in blue and the genes that are targets of TFs in red (containing seed sequences) or green. **B**: Genes containing TFBS and down-regulated can be further divided into two groups: those containing binding sites for both TF and miR-K12-11 (red), and those only containing TFBS but not seed sequences for miR-K12-11 (green).

Putative downstream targets of CEBPβ, E2F1, PAX6, RELA and STAT1 were identified based on screening for corresponding transcription factor binding sites within promoter regions using HMM algorithms [64]. Initially, 3000 to 8000 putative TFBS were catalogued. Genes that were not on the array, or were not expressed in mock transduced cells (i.e. low intensity spots on the array) were omitted. Genes not differentially down regulated in the control vs miRK12-11 were also removed in order to focus specifically on genes that were responsive to ectopic microRNA expression. Due to the spatial and temporal dynamics of gene expression, TF binding is predominantly cell type specific [65]. The DNase-seq data on HUVEC cells (primary endothelial cells) from the ENCODE project enabled identification of active chromatin regions. Genes that did not show DNase hypersensitivity were also filtered from our list of genes with TFBS as they lack TF accessibility. These filtering steps were applied to each of the lists generated from the preliminary prediction results in consideration of the cellular context and the lack of tissue specificity in computational prediction. After filtering, 480 genes were deemed possible targets of CEBPβ, 240 for E2F1, 274 for PAX6, 499 for RELA, and 571 for STAT1. While all of these genes contained TFBS for the corresponding TF, more than 66% of these genes did not contain seed sequence matches for miR-K12-11. Therefore their down-regulation was unlikely to be due to direct targeting by miR-K12-11, but through the repression of the TFs by miR-K12-11. This analysis constructed the extended GRNs of miR-K12-11, including the candidate direct targets of a small number of TFs and hundreds of downstream genes (Figure 5).

Co-occupancy of different TFs on promoters can form distinct functional regulatory complexes in a cell type specific manner. These complexes or regulatory modules are a mechanism especially common to pleiotropic TFs such as E2Fs and STATs [66]. We examined our context specific TFBS prediction, and found that co-localization of multiple TFs on promoters was frequent (Table 2). Putative Co-binding of STAT1 and E2F1 was identified for 92 down-regulated genes (14.96% of down-regulated genes; chi-square test p < 0.05). RELA and STAT1 coocupied particularly frequent (n = 200; 32.52% of the down-regulated genes; chi-square test p <0.001), consistent with data that activation of some genes requires binding of both STAT1 and NFKB [67].

**Table 2:** **Co-binding of multiple TFs on same promoters**

Cobind of	Frequency	Percent
CEBPB X E2F1	69	11.22%
CEBPB X PAX6	128	20.81%
CEBPB X RELA	191	31.06%
CEBPB X STAT1	181	29.43%
E2F1 X PAX6	46	7.48%
E2F1 X RELA	88	14.31%
E2F1 X STAT1	92	14.96%
PAX6 X RELA	115	18.70%
PAX6 X STAT1	93	15.12%
RELA X STAT1	200	32.52%

Percentage was based on 615 down-regulated genes.

A protein-protein interaction (PPI) pair can transmit the expression change of one protein that was repressed by the miRNA to its interacting partner (Figure 1). Combining TFBS with the PPI map provided more details for extending regulatory effects. For this purpose, we assembled the complete human protein interactome from IntAct [26] and BioGrid [27, 28]. The complete interactome contains 173,609 interacting pairs represented by 11,494 genes. The connectivity and the neighbor numbers followed power law distribution (Additional file 2: Figure S3). This comprehensive human PPI network contains all available gene identifiers as the focal genes and all genes that physically bind to each focal gene as its interacting genes. A focal gene and its directly interacting genes were defined as a subnetwork.

**Figure 6:**
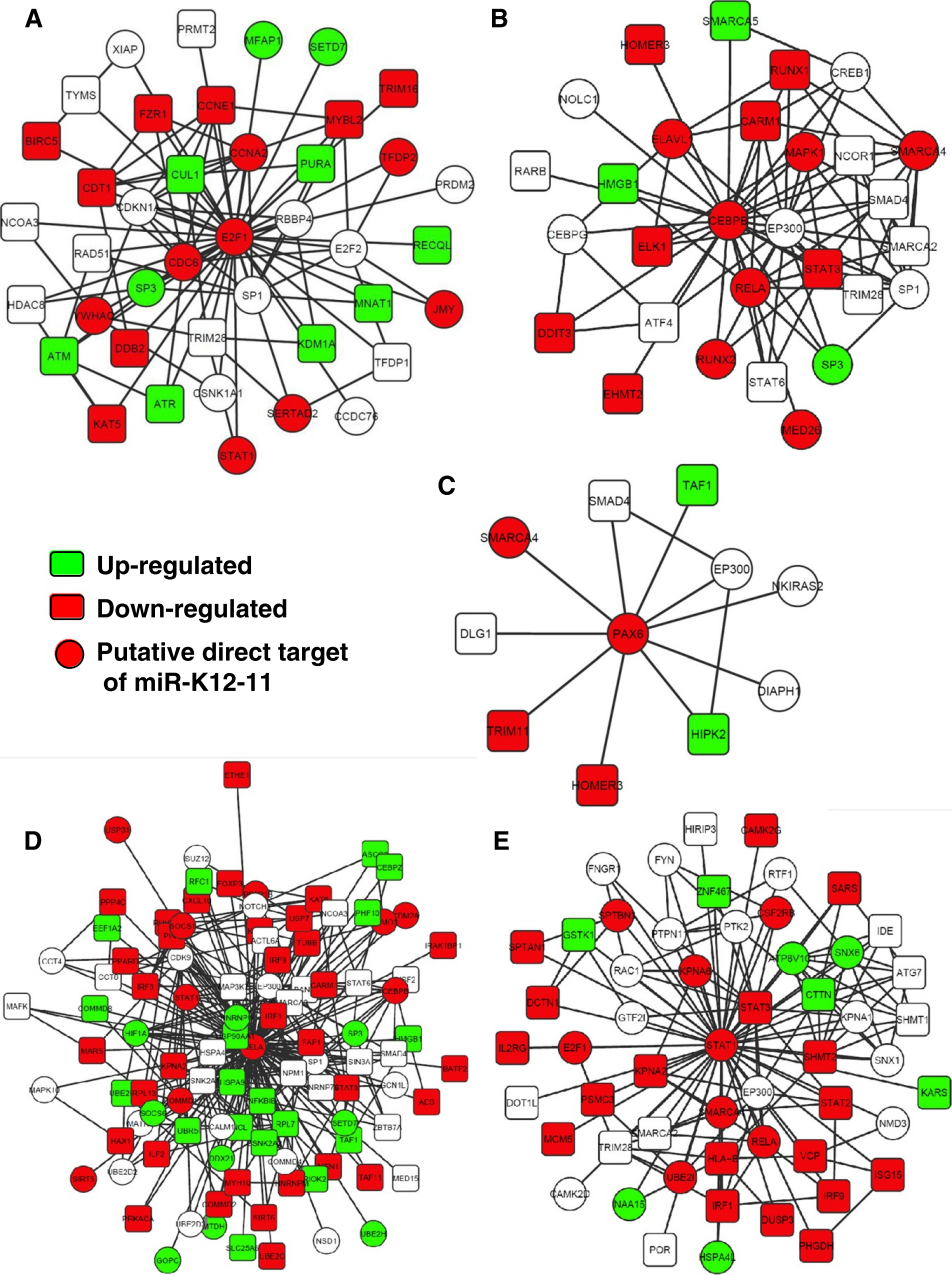
**Change of expression in the interacting genes with the five transcription factors.** Among the genes that directly interact with E2F1 **(A)**, CEBPB **(B)**, PAX6 **(C)**, RELA **(D)** and STAT1 **(E)**, there is an enrichment of down-regulation in accordance with the center node TF genes. Protein interactions, as well as direct targeting of miR-K12-11 (genes of the circles) may contribute to the coordinated down-regulation.

To refine the PPI for the specific biological context in this study, we integrated the curated interactome with our expression data, and removed nodes for genes not on our array and non-expressed genes from the PPI network. For each sub-network consisting of a node and all its interacting genes, the enrichment for down regulated targets of miR-K12-11 was tested. We found that the neighboring genes of E2F1 were enriched with genes down-regulated by miR-K12-11, indicating that the sub-network was targeted (Figure 6). Similar local enrichment for down regulated targets was also identified for non-TF proteins, like the apoptosis effector CASP9 (Additional file 2: Figure S2).

**Figure 7:**
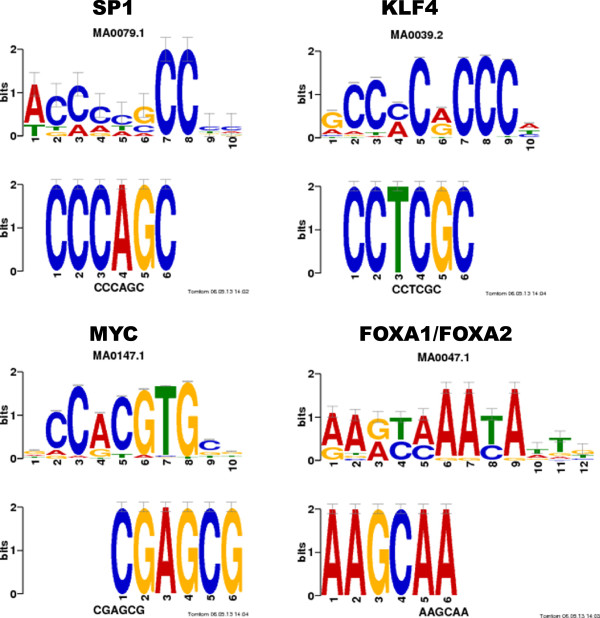
**Motif enrichment analysis from DEGs of BJAB cells.** Motifs identified from the promoter sequences of genes down-regulated by miR-K12-11 in BJAB cells matched the conserved binding motifs of SP1, KLF4 and MYC. SP1 and KLF4 were down-regulated themselves and may therefore relay the regulatory effect to a larger group of genes. For up-regulated genes in response to miR-K12-11, motif of FOXA1 and FOXA2 were identified as putative mediators for the increase of gene expression in BJAB cells.

The degree of expression level changes for the effectors in BJAB cells were more subtle (Table 1). Still, miR-K12-11 overexpression causes expression changes in more than 1000 genes in addition to 197 directly targeted genes. TFs were identified from the putative direct targets, including E2F1, a TF directly targeted by miR-K12-11 also in TIVE cells. To examine TF-dependent regulation affected by miR-K12-11 in BJAB cells, we analyzed the promoter sequences of DEGs using RSAT [68] and TOMTOM [69]. From the set of down-regulated genes, E2F, SP1 and KLF were identified as enriched motifs (Figure 7). These TFs contain the seed sequence of miR-K12-11, supporting their roles as effector genes directly targeted by miR-K12-11. These TFs are also transcriptional activators and the regulatory effect of miR-K12-11 is expected to cause a cascade of repression of transcription.

### miR-K12-11 synergistically regulated multiple signaling pathways to repress the activation of interferon responses

**Figure 8:**
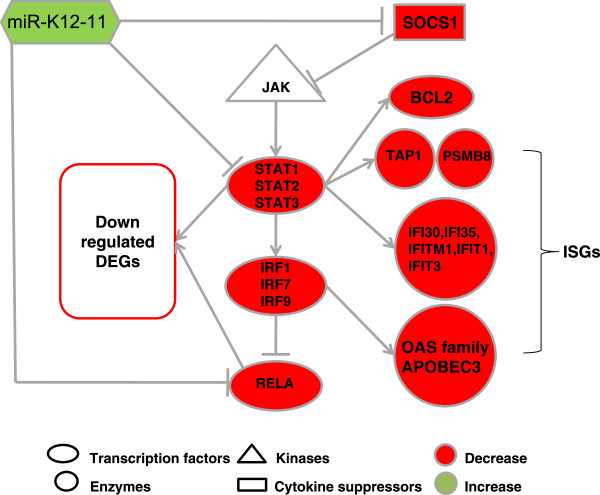
**Interferon responses were repressed via the interplay of multiple signaling pathways and transcription factors.** MiR-K12-11 targeted cytokine receptors and TFs, both of which affected a variety of interferon stimulated genes (ISGs). Through direct and indirect impact, miR-K12-11 is able to modulate the host innate immune response and to help KSHV to establish latency.

MiR-K12-11 also regulates interferon responses and a variety of signaling pathways (Figure 8). Signaling pathways have been suggested as logical targets of miRNA regulation, where small changes in the expression level of upstream genes can affect the signal transduction cascade significantly [70]. Individual miRNAs are able to target several components of a single signaling pathway, as in the cases of miR-8 for Wnt signaling [71], miR-21 for RTK signaling [72, 73] and miR-126 for VEGF signaling [74, 75]. We identified multiple layers of JAK-STAT signaling that were affected by miR-K12-11, with direct targets differing between BJAB and TIVE cells (Additional file 1: Table S4). In BJAB cells, the putative direct targets include the cytokine receptor IFNGR1 (fc > 1.2), which is a confirmed target of miR-155 [76]. In TIVE cells, miR-K12-11 directly targeted SOCS1 (fold change > 1.4, FDR <0.05) and the transcription activator STATs (STAT1 and STAT2 fold change >2 and STAT3 fold change >1.4 FDR < 0.05 for all) (Figure 8; Additional file 1: Table S4).

Interferons are potent cytokines produced in response to viral infection that mediate both innate immune response and subsequent development of adaptive immunity. Modulation of interferon pathways is required to suppress the innate immune response and establish successful latent infection. Along with JAK-STAT signaling, multiple other signaling pathways associated with interferon responses were targeted by miR-K12-11. In TIVE cells, miR-K12-11 targets PTEN and AKT1S1 of the AKT pathway, SKI and SMAD4 of the TGF-β signaling pathway, MYD88 of the TLR-MYD88 pathway which regulates host defense, and RELA of the NF-κB signaling pathway (Additional file 1: Table S4).

The affected signaling pathways are not independent from each other but known to be coordinated through cross-talking [77]. The cooperation of STATs and NF-κB can activate downstream antiviral genes such as the IRFs, a family of transcription factors (TFs) (Additional file 3: Table S5) [67, 78]. IRFs and other TFs such as NF-κB and AP-1 complex (ATF-FOS-JUN) regulate the expression of interferons. Besides their transcriptional activation property, STATs also mediate the IFN response through competition with AP-1 [79]. In BJAB cells, IRF3, ATF1, ATF4 and ATF5 were down-regulated by miR-K12-11 (Additional file 1: Table S4; Additional file 3: Table S5), but not likely through direct binding because they do not contain the seed sequence match sites.

In TIVE cells, a consistent decrease of expression levels was observed for STAT1, STAT2, STAT3, and their transcriptionally regulated genes (Figure 8). RELA and ATF7, which contain the seed sequence of miR-K12-11 and are down regulated are putative direct targets by miR-K12-11 (Additional file 1: Table S4). JUND (member of JUN, protects cell from apoptosis) and multiple IRFs were also down-regulated through indirect effects. The decreased expression of IRF1, IRF7 and IRF9 (also known as p48) may be due to reduced STAT levels since none of these IRFs contain seed sequence matches (Additional file 1: Table S4). While RELA expression is subject to the negative regulation of IRF7, we show that it is directly downregulated by miR-K12-11. A similar functional loop has been reported for miR-155, which by attenuating NF-κB activity, contributes to stabilization of EBV latency [80]. IRF9 can also interact with STAT dimers to form a protein complex to bind promoter sequences [81]. As important TFs, these reduced IRFs likely affected a variety of downstream genes. A number of well characterized interferon stimulated genes (ISGs) such as ISG15, USP18 and the OAS gene family all exhibited significant down-regulation by miR-K12-11, strongly supporting inhibition of interferon responses in endothelial cells (Additional file 2: Table S3; Additional file 1: Table S4).

Liang *et al.* [82] has identified IKKϵ as a miR-K12-11 target in lung cancer cells. Though IKKϵ level was unchanged in this experiment, its downstream effector IRF and NF-κB were reduced. It is likely that miR-K12-11 attenuates IFN signaling by down-regulating multiple possible components, IKKϵ in lung cancer cells, IFNGR1 in B cells, and STAT1 in endothelial cells (Figure 5; Additional file 2: Figure S2). Targeting of these key components not only eliminated the activation of IFN response, but also increased key proliferative and survival signals that are beneficial for KSHV latency establishment.

In addition to miR-K12-11, KSHV expresses homologs to cellular IRFs, that prevent the association of IRFs with their co-activators [83, 84]. The inhibition imposed by miR-K12-11 and vIRF to cellular IRFs may reinforce each other through a feedforward loop. While we cannot estimate the relative contribution of miR-K12-11 versus vIRF signaling, expressing a miRNA comes with the added advantage of not eliciting humoral host immune responses like the protein products do. Other KSHV gene products such as v-cyclin and vIL-6 are also cytokine signaling genes that can block the activity of host homologs [85]. Taken together and in part due to miR-K12-11, KSHV is able to manipulate cell cycle and apoptosis, to evade immune response, and promote proliferation, and survival of infected cells.

## Conclusions

Analyzing GRNs provides insights into the regulatory networks of miRNA regulation that cannot be found by studying single genes. Examining miRNA target genes in the context of cellular GRNs can separate targets that drive phenotypic consequences from non-functional ones. GRNs are highly tissue specific [43, 44, 86, 87], therefore it is imperative to recognize the tissue specificity and define the GRNs of the miRNA only in the relevant cell types. We demonstrated a systems approach to infer the combinatorial GRNs utilized by miR-K12-11 in cellular contexts that are close to KSHV infection *in vivo*. This study included the first target identification of KSHV miRNAs in TIVE cells, a frequently used endothelial cell culture system for studying KSHV infection [88–90].

We found that miR-K12-11 functioned at different hierarchical levels of the GRNs. Putative direct targets of miR-K12-11 were underrepresented in the altered transcriptomes. By targeting a few effector genes, five times more genes were affected beyond direct sequence pairing. Different components, but frequently of common biological pathways, were targeted in BJAB and TIVE cells. There was a preference to targeting TFs, including CEBPβ, PAX6, RELA, and STAT1 in TIVE cells, FOXA, KLF and SP1 in BJAB cells, and E2F1 common to both. Decrease in the TF levels significantly amplified the effect of miR-K12-11 to many more genes downstream, which could potentially result in broad phenotypic effects such as inducing endothelial cell differentiation in the context of KSHV infection. Since viral miRNAs co-evolve with host genes and can be functional orthologs, we found that like its cellular homolog miR-155 [29, 30], miR-K12-11 is also involved in innate and adaptive immune functions by modulating the interferon response and carbohydrate metabolism. Previously validated targets of miR-155 such as CEBPβ and SOCS1 were also identified.

MiR-K12-11 also regulated genes at the middle and bottom of the well-known signaling cascades, like signaling proteins and caspases, and modulated key biological processes like cell cycle control and various signaling pathways, all of which were accomplished by targeting distinct sets of genes within each cell type. Host responses to viral infection, such as innate immunity and apoptosis, are countered by miR-K12-11 and additional viral gene products, enabling the establishment of latency. The multilevel regulation allowed one individual miRNA to profoundly affect the gene expression program to adapt to specific needs.

Finally, the approach we have taken here to identifying miR-K12-11 GRNs can be applied to investigating the viral and cellular miRNAs in different tissues and systems. With an anticipated expansion of genome wide data on short RNA profiles, ChIP, ribonomics, and proteomics in the near future, our strategy could be applied to reveal conditional regulatory pathways in a highly tissue and cell type specific manner.

## Methods

The experimental design allows comparison of miR-155 transduced cells, miR-K12-11 treated cells, and mock transduced cells. The experiment was conducted in four subsequent time periods such that all the experimental conditions were independently repeated.

### Vector system

The foamy virus vector plasmid pCEGFPL was constructed as described before [62]. The gag, pol and env genes are replaced by a miRNA gene following a minimal human cytomegalovirus (CMV) immediate-early promoter at the transcription start site located in the 5′-LTR and a GFP gene as the reporter. The replication ability of the viral vector can be restored by co-transfection with the packaging plasmid pCI env3.5. Recombinant virus vectors expressing miR-155, miR-K12-11 and empty vector without insert as the control were produced by transient cotransfection with Mirus transfection reagent following the manufacturer’s instructions. The supernatant was filtered, concentrated by centrifugation. Resulting foamy viruses were titrated on fresh 293Tand green cells were evaluated for GFP expression using fluorescence microscopy. Notably, empty vectors may lack the control over the non-specific effect of the precursor transcripts but they were able to reduce the off-target effects of a scramble insert.

### Cell culture

BJAB is a Burkitt’s lymphoma human B cell line that is uninfected and Epstein-Barr virus-negative. BJAB cells were grown in culture suspension in complete RPMI medium with 10% fetal bovine serum (FBS). *T*elomerase-*i*mmortalized human umbilical-*v*ein *e*ndothelial (TIVE) cells [88] have been specially developed for the purpose of studying the effects of KHSV latent infection in endothelial cells. TIVE cells are adherent cells grown in Medium 199 supplemented with 20% FBS and 60 μg/mL Endothelial Cell Growth Factor (ECGF).

### Transduction and validation

TIVE and BJAB cells were retrovirally transduced at two levels of Multiplicity of Infection (MOI): 1 and 10. 72 hr post transduction, positive cells were sorted according to their GFP signal. Cells were aliquoted in 1 million cells per tube and frozen down in liquid nitrogen. Empty vectors without miRNA expression cassettes were used for mock transduction to control for the impact of retroviral integration on the cellular transcriptomes. The aim of the freezing is to synchronize the growth status of the cells across samples, and to reduce noise to microarray profiling. Later, cells were removed from liquid nitrogen and grown for the same number of passages. RNA was extracted using the RNA-Bee reagent according to the manufacturer’s instructions. The quantity and quality of RNA was confirmed by NanoDrop spectrometer and agarose gel electrophoresis. The integrity of total RNA was assessed with Agilent Bioanalyzer. Expression of miRNAs was examined using TaqMan qPCR. Expression levels of miR-155 and miR-K12-11 were normalized to RNU66 levels. The MOI did not result in differences in miRNA expression levels. Therefore, all samples were treated as biological replicates.

### Microarray analysis

For each HG-133 plus 2.0 chip, 200 ng RNA was used as the starting material. RNA was synthesized and labeled using GeneChip® 3′ IVT Express Kit and chips were hybridized according to manufacturer instructions (Affymetrix). Raw data (cell intensity files, CEL) were summarized using Affymetrix Expression Console software (v1.1). Chips were examined for successful hybridization by ensuring that the marginal distribution of all slides was similar. Samples were compared for the global effect of miRNA treatment at a population level using principal component analysis [91]. Probe sets were flagged as ‘absent’ if they were absent according to Affymetrix probe detection algorithm (Affymetrix Statistical Algorithms Description Document. http://media.affymetrix.com/support/technical/whitepapers/sadd_whitepaper.pdf) in more than half of the samples. The data were deposited in the GEO database with accession number GSE59412.

The following model was fit Y_ij_ = μ + α_i_ + ϵ_ij_ , where Y_ij_ is the difference of the log2 signals for each probe set between the miRNA transduced and control vector for the i^th^ condition and the j^th^ replicate; μ is the difference for the overall expression mean. ϵ_ij_ ~ N (0, σ_i_^2^). The signal differences between miRNA transduced samples and their corresponding control samples were used as this paired design reflects the experimental design. The test of the null hypothesis that α_i_ =0 is a direct test of the miRNA condition. F tests for each of the miRNA conditions (miR-155 in BJAB, miR-K12-11 in BJAB, miR-K12-11 in TIVE) were conducted. An FDR of 0.05 was used to determine statistical significance for the probe set [92].

The probe sets were annotated by comparing the genome positions of human genes and of probe set hits. A gene was considered differentially expressed (DEG) when at least one probe set was significant. The change in expression levels was the difference in the mean of all probe sets between treatment and control. DEGs were examined for potential functional groups by enrichment analysis [93]. Enriched Gene Ontology terms [94] of the DEGs and known biological pathways were compared using Fisher’s exact test.

### Identification of direct miRNA targets

To increase the specificity of our GRN inference, we focused on the canonical targets, for which a range of targeting rules have been defined and most prediction algorithms are developed. Even so, our analysis and previous reports [19, 95] found the lack of concordance across the miRNA target prediction of different algorithms. This is the result of using different training set of target genes when the algorithms were developed. A comprehensive list of putative targets of miR-155/miR-K12-11 was created by using the union of target prediction from multiple algorithms: EMBL-EBI mirBase [96], TargetScan [21], PITA [97], DIANA [98], miRDB [99], RNA22 [100], mirWalk [101], mirZ [102] and PicTar [103]. In addition, SylArray [104] was used to identify enrichment of miRNA seed sequence matches. The predicted targets were also compared to validated target genes in the literature.

### Identification of transcription factor regulation

A list of human transcriptional factor (TF) genes was obtained from the JASPAR database [105] and a TF census study [106]. DEGs on this list as well on the miRNA target list were examined in detail for expression changes and biological implications, as they were the primary targets of the miRNA. We used MAPPER [64, 107], which uses binding site information from TRANSFAC and JASPAR databases derived Hidden Markov Models, to detect putative transcription factor binding sites (TFBS). Genes containing TFBS within the upstream 2 kb region of transcription start sites were identified as genes that might be under TF regulation.

For DEGs with the same direction of expression change, enriched motifs in their promoter regions were identified using RSAT oligo analysis [68]. The motifs were compared to the binding motifs of TFs using the TOMTOM program of the MEME suite [69]. Motifs identified from up- and down-regulated set of DEGs were compared, and unique motifs for each set were identified. Additional evidence for TF regulation was obtained from literature search and the Transcriptional Regulatory Element Database (TRED) [108]. ChIP-seq (measuring DNA-protein interaction) and DNase-seq (measuring DNA accessibility to regulatory proteins) profiles of the ENCODE project [65] from corresponding cell types were used to constrain the TF regulated genes to be tissue specific.

### Identification of signaling genes

Human signaling pathway data was obtained from the National Cancer Institute Pathway Interaction Database (NCI PID) [109], which is a manually curated collection of biomolecular interactions and key cellular processes assembled into signaling pathways. NCI PID holds 128 pathways including 47 sub-networks. All subnetworks with their parent networks were combined to generate the set of signaling pathways. Pathways curated in the BioCarta database (http://www.biocarta.com/) were used for cross-referencing to reduce ambiguity. In addition, all pathways that have more than one predicted microRNA target gene were kept, leading to a final data set of 79 human signaling pathways containing 1573 unique human proteins. The database also provides information on subcellular location terms from the Gene Ontology Consortium. Process type information was extracted for each biological process, which can be input, output, positive or negative regulator. In total, there are 1120 interactions of which 765 are activating, 74 inhibiting and 281 proteins acting as activators as well as inhibitors.

### Identification of functional interaction

A binary interactome was assembled enabling an overview of all physical interactions that can occur between human proteins. Gene association data were downloaded from GeneRIF (Gene References into Function) database at NCBI [110] and the IntAct database [26] at EBI on Febuary 28 2011. The interactions in GeneRIF are sourced from Bind [111, 112], BioGrid [27, 28], EcoCyc [113], and HPRD [114]. The IntAct database includes interactions from literature curation at EBI as well as user submission. Only protein-protein interaction data for human was retained. The formatted data contain a list of focal genes that covers all available values of gene identifiers, the interacting genes for each focal gene, the detection method and the source of the interaction. Secondary interactions are derived from the interactions of the genes identified as interactors of the initial focal gene.

The human PPI networks were plotted as undirected graphs, where the nodes are proteins and two nodes are connected by an undirected edge if the corresponding proteins physically bind to each other. DEGs were mapped to the interactomes to identify the interactants of the indirect targets. The expression levels of genes belonging to the map were examined and absent genes were removed. Up- and down-regulated DEGs were flagged to display in different colors. A focal gene and its neighboring genes were defined as a subnetwork. The percentage of DEGs in the subnetwork for each focal gene was calculated. If DEGs were present more often than in the experiment as a whole, the focal gene was identified as an enriched regulator and its subnetwork was considered as responsive. GO enrichment was also examined on the enriched regulators, to determine if transcriptionally regulated sub-networks shared GO terms indicative of known or related biological functions. The subnetworks were viewed in Cytoscape [115, 116] for active biological pathways.

## Electronic supplementary material

Additional file 1: Table S4: Overview of all differentially expressed genes. (XLSX 1 MB)

Additional file 2: **Supplemental tables (Table S1-S3) and figures (Figure S1-S3).** (PDF 276 KB)

Additional file 3: Table S5: Results of transcription factor binding prediction. (XLSX 12 MB)

## References

[1] Chang Y, Cesarman E, Pessin MS, Lee F, Culpepper J, Knowles DM, Moore PS (1994). Identification of herpesvirus-like DNA sequences in AIDS-associated Kaposi’s sarcoma. Science.

[2] Cesarman E, Knowles DM (1999). The role of Kaposi’s sarcoma-associated herpesvirus (KSHV/HHV-8) in lymphoproliferative diseases. Semin Cancer Biol.

[3] Soulier J, Grollet L, Oksenhendler E, Cacoub P, Cazals-Hatem D, Babinet P, d’Agay MF, Clauvel JP, Raphael M, Degos L, Sigaux F (1995). Kaposi’s sarcoma-associated herpesvirus-like DNA sequences in multicentric Castleman’s disease. Blood.

[4] Dittmer D, Lagunoff M, Renne R, Staskus K, Haase A, Ganem D (1998). A cluster of latently expressed genes in Kaposi’s sarcoma-associated herpesvirus. J Virol.

[5] Renne R, Lagunoff M, Zhong W, Ganem D (1996). The size and conformation of Kaposi’s sarcoma-associated herpesvirus (human herpesvirus 8) DNA in infected cells and virions. J Virol.

[6] Zhong W, Wang H, Herndier B, Ganem D (1996). Restricted expression of Kaposi sarcoma-associated herpesvirus (human herpesvirus 8) genes in Kaposi sarcoma. Proc Natl Acad Sci U S A.

[7] Baek D, Villen J, Shin C, Camargo FD, Gygi SP, Bartel DP (2008). The impact of microRNAs on protein output. Nature.

[8] Selbach M, Schwanhausser B, Thierfelder N, Fang Z, Khanin R, Rajewsky N (2008). Widespread changes in protein synthesis induced by microRNAs. Nature.

[9] Farh KK-H, Grimson A, Jan C, Lewis BP, Johnston WK, Lim LP, Burge CB, Bartel DP (2005). The widespread impact of mammalian MicroRNAs on mRNA repression and evolution. Science.

[10] Eulalio A, Huntzinger E, Nishihara T, Rehwinkel J, Fauser M, Izaurralde E (2009). Deadenylation is a widespread effect of miRNA regulation. RNA.

[11] Guo H, Ingolia NT, Weissman JS, Bartel DP (2010). Mammalian microRNAs predominantly act to decrease target mRNA levels. Nature.

[12] Lewis BP, Burge CB, Bartel DP (2005). Conserved seed pairing, often flanked by adenosines, indicates that thousands of human genes are microRNA targets. Cell.

[13] Lim LP, Lau NC, Garrett-Engele P, Grimson A, Schelter JM, Castle J, Bartel DP, Linsley PS, Johnson JM (2005). Microarray analysis shows that some microRNAs downregulate large numbers of target mRNAs. Nature.

[14] Stark A, Brennecke J, Russell RB, Cohen SM (2003). Identification of Drosophila MicroRNA targets. PLoS Biol.

[15] Lewis BP, Shih IH, Jones-Rhoades MW, Bartel DP, Burge CB (2003). Prediction of mammalian microRNA targets. Cell.

[16] Grimson A, Farh KK, Johnston WK, Garrett-Engele P, Lim LP, Bartel DP (2007). MicroRNA targeting specificity in mammals: determinants beyond seed pairing. Mol Cell.

[17] Bartel DP (2009). MicroRNAs: target recognition and regulatory functions. Cell.

[18] Mazière P, Enright AJ (2007). Prediction of microRNA targets. Drug Discov Today.

[19] Alexiou P, Maragkakis M, Papadopoulos GL, Reczko M, Hatzigeorgiou AG (2009). Lost in translation: an assessment and perspective for computational microRNA target identification. Bioinformatics.

[20] Zhu Y, Haecker I, Yang Y, Gao S-J, Renne R (2013). γ-Herpesvirus-encoded miRNAs and their roles in viral biology and pathogenesis. Curr Opin Virol.

[21] Friedman RC, Farh KK, Burge CB, Bartel DP (2009). Most mammalian mRNAs are conserved targets of microRNAs. Genome Res.

[22] Mukherji S, Ebert MS, Zheng GX, Tsang JS, Sharp PA, van Oudenaarden A (2011). MicroRNAs can generate thresholds in target gene expression. Nat Genet.

[23] Graur D, Zheng Y, Price N, Azevedo RB, Zufall RA, Elhaik E (2013). On the immortality of television sets: “function” in the human genome according to the evolution-free gospel of ENCODE. Genome Biol Evol.

[24] Park SM, Gaur AB, Lengyel E, Peter ME (2008). The miR-200 family determines the epithelial phenotype of cancer cells by targeting the E-cadherin repressors ZEB1 and ZEB2. Genes Dev.

[25] Ideker T, Thorsson V, Ranish JA, Christmas R, Buhler J, Eng JK, Bumgarner R, Goodlett DR, Aebersold R, Hood L (2001). Integrated genomic and proteomic analyses of a systematically perturbed metabolic network. Science.

[26] Kerrien S, Alam-Faruque Y, Aranda B, Bancarz I, Bridge A, Derow C, Dimmer E, Feuermann M, Friedrichsen A, Huntley R, Kohler C, Khadake J, Leroy C, Liban A, Lieftink C, Montecchi-Palazzi L, Orchard S, Risse J, Robbe K, Roechert B, Thorneycroft D, Zhang Y, Apweiler R, Hermjakob H (2007). IntAct–open source resource for molecular interaction data. Nucleic Acids Res.

[27] Breitkreutz BJ, Stark C, Reguly T, Boucher L, Breitkreutz A, Livstone M, Oughtred R, Lackner DH, Bahler J, Wood V, Dolinski K, Tyers M (2008). The BioGRID Interaction Database: 2008 Update. Nucleic Acids Res.

[28] Stark C, Breitkreutz B-J, Reguly T, Boucher L, Breitkreutz A, Tyers M (2006). BioGRID: a general repository for interaction datasets. Nucleic Acids Res.

[29] Gottwein E, Mukherjee N, Sachse C, Frenzel C, Majoros WH, Chi JT, Braich R, Manoharan M, Soutschek J, Ohler U, Cullen BR (2007). A viral microRNA functions as an orthologue of cellular miR-155. Nature.

[30] Skalsky RL, Samols MA, Plaisance KB, Boss IW, Riva A, Lopez MC, Baker HV, Renne R (2007). Kaposi’s sarcoma-associated herpesvirus encodes an ortholog of miR-155. J Virol.

[31] Moffett H, Novina C (2007). A small RNA makes a Bic difference. Genome Biol.

[32] Thai TH, Calado DP, Casola S, Ansel KM, Xiao C, Xue Y, Murphy A, Frendewey D, Valenzuela D, Kutok JL, Schmidt-Supprian M, Rajewsky N, Yancopoulos G, Rao A, Rajewsky K (2007). Regulation of the germinal center response by microRNA-155. Science.

[33] Rodriguez A, Vigorito E, Clare S, Warren MV, Couttet P, Soond DR, van Dongen S, Grocock RJ, Das PP, Miska EA, Vetrie D, Okkenhaug K, Enright AJ, Dougan G, Turner M, Bradley A (2007). Requirement of bic/microRNA-155 for normal immune function. Science.

[34] Costinean S, Zanesi N, Pekarsky Y, Tili E, Volinia S, Heerema N, Croce CM (2006). Pre-B cell proliferation and lymphoblastic leukemia/high-grade lymphoma in E(mu)-miR155 transgenic mice. Proc Natl Acad Sci U S A.

[35] Eis PS, Tam W, Sun L, Chadburn A, Li Z, Gomez MF, Lund E, Dahlberg JE (2005). Accumulation of miR-155 and BIC RNA in human B cell lymphomas. Proc Natl Acad Sci U S A.

[36] Kluiver J, Poppema S, de Jong D, Blokzijl T, Harms G, Jacobs S, Kroesen BJ, van den Berg A (2005). BIC and miR-155 are highly expressed in Hodgkin, primary mediastinal and diffuse large B cell lymphomas. J Pathol.

[37] O’Connell RM, Rao DS, Chaudhuri AA, Boldin MP, Taganov KD, Nicoll J, Paquette RL, Baltimore D (2008). Sustained expression of microRNA-155 in hematopoietic stem cells causes a myeloproliferative disorder. J Exp Med.

[38] van den Berg A, Kroesen BJ, Kooistra K, de Jong D, Briggs J, Blokzijl T, Jacobs S, Kluiver J, Diepstra A, Maggio E, Poppema S (2003). High expression of B-cell receptor inducible gene BIC in all subtypes of Hodgkin lymphoma. Genes Chromosomes Cancer.

[39] Jeyapalan Z, Deng Z, Shatseva T, Fang L, He C, Yang BB (2011). Expression of CD44 3′-untranslated region regulates endogenous microRNA functions in tumorigenesis and angiogenesis. Nucleic Acids Res.

[40] Poliseno L, Salmena L, Zhang J, Carver B, Haveman WJ, Pandolfi PP (2010). A coding-independent function of gene and pseudogene mRNAs regulates tumour biology. Nature.

[41] Tay Y, Kats L, Salmena L, Weiss D, Tan SM, Ala U, Karreth F, Poliseno L, Provero P, Di Cunto F, Lieberman J, Rigoutsos I, Pandolfi PP (2011). Coding-independent regulation of the tumor suppressor PTEN by competing endogenous mRNAs. Cell.

[42] Sumazin P, Yang X, Chiu HS, Chung WJ, Iyer A, Llobet-Navas D, Rajbhandari P, Bansal M, Guarnieri P, Silva J, Califano A (2011). An extensive microRNA-mediated network of RNA-RNA interactions regulates established oncogenic pathways in glioblastoma. Cell.

[43] Gerstein MB, Kundaje A, Hariharan M, Landt SG, Yan KK, Cheng C, Mu XJ, Khurana E, Rozowsky J, Alexander R, Min R, Alves P, Abyzov A, Addleman N, Bhardwaj N, Boyle AP, Cayting P, Charos A, Chen DZ, Cheng Y, Clarke D, Eastman C, Euskirchen G, Frietze S, Fu Y, Gertz J, Grubert F, Harmanci A, Jain P, Kasowski M (2012). Architecture of the human regulatory network derived from ENCODE data. Nature.

[44] Thurman RE, Rynes E, Humbert R, Vierstra J, Maurano MT, Haugen E, Sheffield NC, Stergachis AB, Wang H, Vernot B, Garg K, John S, Sandstrom R, Bates D, Boatman L, Canfield TK, Diegel M, Dunn D, Ebersol AK, Frum T, Giste E, Johnson AK, Johnson EM, Kutyavin T, Lajoie B, Lee BK, Lee K, London D, Lotakis D, Neph S (2012). The accessible chromatin landscape of the human genome. Nature.

[45] Jiang S, Zhang HW, Lu MH, He XH, Li Y, Gu H, Liu MF, Wang ED (2010). MicroRNA-155 functions as an OncomiR in breast cancer by targeting the suppressor of cytokine signaling 1 gene. Cancer Res.

[46] Demorest ZL, Li M, Harris RS (2011). Phosphorylation directly regulates the intrinsic DNA cytidine deaminase activity of activation-induced deaminase and APOBEC3G protein. J Biol Chem.

[47] Dorsett Y, McBride KM, Jankovic M, Gazumyan A, Thai TH, Robbiani DF, Di Virgilio M, Reina San-Martin B, Heidkamp G, Schwickert TA, Eisenreich T, Rajewsky K, Nussenzweig MC (2008). MicroRNA-155 suppresses activation-induced cytidine deaminase-mediated Myc-Igh translocation. Immunity.

[48] Teng G, Hakimpour P, Landgraf P, Rice A, Tuschl T, Casellas R, Papavasiliou FN (2008). MicroRNA-155 is a negative regulator of activation-induced cytidine deaminase. Immunity.

[49] Delgado T, Carroll PA, Punjabi AS, Margineantu D, Hockenbery DM, Lagunoff M (2010). Induction of the Warburg effect by Kaposi’s sarcoma herpesvirus is required for the maintenance of latently infected endothelial cells. Proc Natl Acad Sci U S A.

[50] Warburg O (1956). On the origin of cancer cells. Science.

[51] Warburg O, Posener K, Negelein E (1924). Ueber den Stoffwechsel der Tumoren. Biochem Z.

[52] Haecker I, Gay LA, Yang Y, Hu J, Morse AM, McIntyre LM, Renne R (2012). Ago HITS-CLIP expands understanding of Kaposi’s sarcoma-associated herpesvirus miRNA function in primary effusion lymphomas. PLoS Pathog.

[53] Rhoades MW, Reinhart BJ, Lim LP, Burge CB, Bartel B, Bartel DP (2002). Prediction of plant microRNA targets. Cell.

[54] Enright AJ, John B, Gaul U, Tuschl T, Sander C, Marks DS (2003). MicroRNA targets in Drosophila. Genome Biol.

[55] Tu K, Yu H, Hua Y-J, Li Y-Y, Liu L, Xie L, Li Y-X (2009). Combinatorial network of primary and secondary microRNA-driven regulatory mechanisms. Nucleic Acids Res.

[56] Liang H, Li WH (2007). MicroRNA regulation of human protein interaction network. RNA.

[57] Shalgi R, Lieber D, Oren M, Pilpel Y (2007). Global and local architecture of the mammalian microRNA-transcription factor regulatory network. PLoS Comput Biol.

[58] Martinez NJ, Ow MC, Barrasa MI, Hammell M, Sequerra R, Doucette-Stamm L, Roth FP, Ambros VR, Walhout AJ (2008). A C. elegans genome-scale microRNA network contains composite feedback motifs with high flux capacity. Genes Dev.

[59] Cui Q, Yu Z, Pan Y, Purisima EO, Wang E (2007). MicroRNAs preferentially target the genes with high transcriptional regulation complexity. Biochem Biophys Res Commun.

[60] Kabnick KS, Housman DE (1988). Determinants that contribute to cytoplasmic stability of human c-fos and beta-globin mRNAs are located at several sites in each mRNA. Mol Cell Biol.

[61] Yeilding NM, Rehman MT, Lee WM (1996). Identification of sequences in c-myc mRNA that regulate its steady-state levels. Mol Cell Biol.

[62] Boss IW, Nadeau PE, Abbott JR, Yang Y, Mergia A, Renne R (2011). A Kaposi’s sarcoma-associated herpesvirus-encoded ortholog of MicroRNA miR-155 induces human splenic B-cell expansion in NOD/LtSz-scid IL2Rγnull mice. J Virol.

[63] Yin Q, Wang X, Fewell C, Cameron J, Zhu H, Baddoo M, Lin Z, Flemington EK (2010). MicroRNA miR-155 inhibits bone morphogenetic protein (BMP) signaling and BMP-mediated Epstein-Barr virus reactivation. J Virol.

[64] Riva A (2012). The MAPPER2 Database: a multi-genome catalog of putative transcription factor binding sites. Nucleic Acids Res.

[65] Neph S, Vierstra J, Stergachis AB, Reynolds AP, Haugen E, Vernot B, Thurman RE, John S, Sandstrom R, Johnson AK, Maurano MT, Humbert R, Rynes E, Wang H, Vong S, Lee K, Bates D, Diegel M, Roach V, Dunn D, Neri J, Schafer A, Hansen RS, Kutyavin T, Giste E, Weaver M, Canfield T, Sabo P, Zhang M, Balasundaram G (2012). An expansive human regulatory lexicon encoded in transcription factor footprints. Nature.

[66] Kiuchi N, Nakajima K, Ichiba M, Fukada T, Narimatsu M, Mizuno K, Hibi M, Hirano T (1999). STAT3 is required for the gp130-mediated full activation of the c-myc gene. J Exp Med.

[67] Li Q, Verma IM (2002). NF-kappaB regulation in the immune system. Nat Rev Immunol.

[68] Thomas-Chollier M, Defrance M, Medina-Rivera A, Sand O, Herrmann C, Thieffry D, van Helden J (2011). RSAT 2011: regulatory sequence analysis tools. Nucleic Acids Res.

[69] Bailey TL, Boden M, Buske FA, Frith M, Grant CE, Clementi L, Ren J, Li WW, Noble WS (2009). MEME Suite: tools for motif discovery and searching. Nucleic Acids Res.

[70] Inui M, Martello G, Piccolo S (2010). MicroRNA control of signal transduction. Nat Rev Mol Cell Biol.

[71] Kennell JA, Gerin I, MacDougald OA, Cadigan KM (2008). The microRNA miR-8 is a conserved negative regulator of Wnt signaling. Proc Natl Acad Sci U S A.

[72] Meng F, Henson R, Wehbe-Janek H, Ghoshal K, Jacob ST, Patel T (2007). MicroRNA-21 regulates expression of the PTEN tumor suppressor gene in human hepatocellular cancer. Gastroenterology.

[73] Thum T, Gross C, Fiedler J, Fischer T, Kissler S, Bussen M, Galuppo P, Just S, Rottbauer W, Frantz S, Castoldi M, Soutschek J, Koteliansky V, Rosenwald A, Basson MA, Licht JD, Pena JT, Rouhanifard SH, Muckenthaler MU, Tuschl T, Martin GR, Bauersachs J, Engelhardt S (2008). MicroRNA-21 contributes to myocardial disease by stimulating MAP kinase signalling in fibroblasts. Nature.

[74] Fish JE, Santoro MM, Morton SU, Yu S, Yeh RF, Wythe JD, Ivey KN, Bruneau BG, Stainier DY, Srivastava D (2008). miR-126 regulates angiogenic signaling and vascular integrity. Dev Cell.

[75] Kuhnert F, Mancuso MR, Hampton J, Stankunas K, Asano T, Chen CZ, Kuo CJ (2008). Attribution of vascular phenotypes of the murine Egfl7 locus to the microRNA miR-126. Development.

[76] Banerjee A, Schambach F, DeJong CS, Hammond SM, Reiner SL (2010). Micro-RNA-155 inhibits IFN-gamma signaling in CD4+ T cells. Eur J Immunol.

[77] Shuai K, Liu B (2003). Regulation of JAK-STAT signalling in the immune system. Nat Rev Immunol.

[78] Honda K, Yanai H, Negishi H, Asagiri M, Sato M, Mizutani T, Shimada N, Ohba Y, Takaoka A, Yoshida N, Taniguchi T (2005). IRF-7 is the master regulator of type-I interferon-dependent immune responses. Nature.

[79] Wang G, Wang Y, Teng M, Zhang D, Li L, Liu Y (2010). Signal transducers and activators of transcription-1 (STAT1) regulates microRNA transcription in interferon gamma-stimulated HeLa cells. PLoS One.

[80] Lu F, Weidmer A, Liu CG, Volinia S, Croce CM, Lieberman PM (2008). Epstein-Barr virus-induced miR-155 attenuates NF-kappaB signaling and stabilizes latent virus persistence. J Virol.

[81] Dunn JJ, McCorkle SR, Everett L, Anderson CW (2007). Paired-end genomic signature tags: a method for the functional analysis of genomes and epigenomes. Genet Eng (NY).

[82] Liang D, Gao Y, Lin X, He Z, Zhao Q, Deng Q, Lan K (2011). A human herpesvirus miRNA attenuates interferon signaling and contributes to maintenance of viral latency by targeting IKKvarepsilon. Cell Res.

[83] Lin R, Genin P, Mamane Y, Sgarbanti M, Battistini A, Harrington WJ, Barber GN, Hiscott J (2001). HHV-8 encoded vIRF-1 represses the interferon antiviral response by blocking IRF-3 recruitment of the CBP/p300 coactivators. Oncogene.

[84] Joo CH, Shin YC, Gack M, Wu L, Levy D, Jung JU (2007). Inhibition of interferon regulatory factor 7 (IRF7)-mediated interferon signal transduction by the Kaposi’s sarcoma-associated herpesvirus viral IRF homolog vIRF3. J Virol.

[85] Damania B (2004). Modulation of cell signaling pathways by Kaposi's sarcoma-associated herpesvirus (KSHVHHV-8). Cell Biochem Biophys.

[86] Cheng C, Alexander R, Min R, Leng J, Yip KY, Rozowsky J, Yan KK, Dong X, Djebali S, Ruan Y, Davis CA, Carninci P, Lassman T, Gingeras TR, Guigó R, Birney E, Weng Z, Snyder M, Gerstein M (2012). Understanding transcriptional regulation by integrative analysis of transcription factor binding data. Genome Res.

[87] Paik YK, Hancock WS (2012). Uniting ENCODE with genome-wide proteomics. Nat Biotechnol.

[88] An FQ, Folarin HM, Compitello N, Roth J, Gerson SL, McCrae KR, Fakhari FD, Dittmer DP, Renne R (2006). Long-term-infected telomerase-immortalized endothelial cells: a model for Kaposi’s sarcoma-associated herpesvirus latency in vitro and in vivo. J Virol.

[89] Roy D, Sin SH, Lucas A, Venkataramanan R, Wang L, Eason A, Chavakula V, Hilton IB, Tamburro KM, Damania B, Dittmer DP (2013). mTOR inhibitors block Kaposi sarcoma growth by inhibiting essential autocrine growth factors and tumor angiogenesis. Cancer Res.

[90] Di Bartolo DL, Cannon M, Liu YF, Renne R, Chadburn A, Boshoff C, Cesarman E (2008). KSHV LANA inhibits TGF-beta signaling through epigenetic silencing of the TGF-beta type II receptor. Blood.

[91] Johnson RA, Wichern DW (1992). Applied Multivariate Statistical Analysis.

[92] Benjamini Y, Hochberg Y (1995). Controlling the false discovery rate: a practical and powerful approach to multiple testing. J Royal Stat Soc B (Method).

[93] Mootha VK, Lindgren CM, Eriksson K-F, Subramanian A, Sihag S, Lehar J, Puigserver P, Carlsson E, Ridderstrale M, Laurila E, Houstis N, Daly MJ, Patterson N, Mesirov JP, Golub TR, Tamayo P, Spiegelman B, Lander ES, Hirschhorn JN, Altshuler D, Groop LC (2003). PGC-1[alpha]-responsive genes involved in oxidative phosphorylation are coordinately downregulated in human diabetes. Nat Genet.

[94] Subramanian A, Tamayo P, Mootha VK, Mukherjee S, Ebert BL, Gillette MA, Paulovich A, Pomeroy SL, Golub TR, Lander ES, Mesirov JP (2005). Gene set enrichment analysis: A knowledge-based approach for interpreting genome-wide expression profiles. Proc Natl Acad Sci U S A.

[95] Ritchie W, Flamant S, Rasko JEJ (2009). Predicting microRNA targets and functions: traps for the unwary. Nat Meth.

[96] Betel D, Wilson M, Gabon A, Marks DS, Sander C (2008). The microRNA.org resource: targets and expression. Nucleic Acids Res.

[97] Kertesz M, Iovino N, Unnerstall U, Gaul U, Segal E (2007). The role of site accessibility in microRNA target recognition. Nat Genet.

[98] Kiriakidou M, Nelson PT, Kouranov A, Fitziev P, Bouyioukos C, Mourelatos Z, Hatzigeorgiou A (2004). A combined computational-experimental approach predicts human microRNA targets. Genes Dev.

[99] Wang X (2008). miRDB: a microRNA target prediction and functional annotation database with a wiki interface. RNA.

[100] Miranda KC, Huynh T, Tay Y, Ang YS, Tam WL, Thomson AM, Lim B, Rigoutsos I (2006). A pattern-based method for the identification of MicroRNA binding sites and their corresponding heteroduplexes. Cell.

[101] Dweep H, Sticht C, Pandey P, Gretz N (2011). miRWalk–database: prediction of possible miRNA binding sites by “walking” the genes of three genomes. J Biomed Inform.

[102] Gaidatzis D, van Nimwegen E, Hausser J, Zavolan M (2007). Inference of miRNA targets using evolutionary conservation and pathway analysis. BMC Bioinformatics.

[103] Krek A, Grun D, Poy MN, Wolf R, Rosenberg L, Epstein EJ, MacMenamin P, da Piedade I, Gunsalus KC, Stoffel M, Rajewsky N (2005). Combinatorial microRNA target predictions. Nat Genet.

[104] Bartonicek N, Enright AJ (2010). SylArray: a web server for automated detection of miRNA effects from expression data. Bioinformatics.

[105] Sandelin A, Alkema W, Engström P, Wasserman WW, Lenhard B (2004). JASPAR: an open‒access database for eukaryotic transcription factor binding profiles. Nucleic Acids Res.

[106] Vaquerizas JM, Kummerfeld SK, Teichmann SA, Luscombe NM (2009). A census of human transcription factors: function, expression and evolution. Nat Rev Genet.

[107] Marinescu VD, Kohane IS, Riva A (2005). The MAPPER database: a multi-genome catalog of putative transcription factor binding sites. Nucleic Acids Res.

[108] Jiang C, Xuan Z, Zhao F, Zhang MQ (2007). TRED: a transcriptional regulatory element database, new entries and other development. Nucleic Acids Res.

[109] Schaefer CF, Anthony K, Krupa S, Buchoff J, Day M, Hannay T, Buetow KH (2009). PID: the pathway interaction database. Nucleic Acids Res.

[110] Benson DA, Karsch-Mizrachi I, Lipman DJ, Ostell J, Wheeler DL (2005). GenBank. Nucleic Acids Res.

[111] Bader GD, Hogue CWV (2000). BIND–a data specification for storing and describing biomolecular interactions, molecular complexes and pathways. Bioinformatics.

[112] Bader GD, Donaldson I, Wolting C, Ouellette BFF, Pawson T, Hogue CWV (2001). BIND–The Biomolecular Interaction Network Database. Nucleic Acids Res.

[113] Karp PD, Riley M, Saier M, Paulsen IT, Paley SM, Pellegrini-Toole A (2000). The EcoCyc and MetaCyc databases. Nucleic Acids Res.

[114] Peri S, Navarro JD, Kristiansen TZ, Amanchy R, Surendranath V, Muthusamy B, Gandhi TKB, Chandrika KN, Deshpande N, Suresh S, Rashmi BP, Shanker K, Padma N, Niranjan V, Harsha HC, Talreja N, Vrushabendra BM, Ramya MA, Yatish AJ, Joy M, Shivashankar HN, Kavitha MP, Menezes M, Choudhury DR, Ghosh N, Saravana R, Chandran S, Mohan S, Jonnalagadda CK, Prasad CK (2004). Human protein reference database as a discovery resource for proteomics. Nucleic Acids Res.

[115] Cline MS, Smoot M, Cerami E, Kuchinsky A, Landys N, Workman C, Christmas R, Avila-Campilo I, Creech M, Gross B, Hanspers K, Isserlin R, Kelley R, Killcoyne S, Lotia S, Maere S, Morris J, Ono K, Pavlovic V, Pico AR, Vailaya A, Wang PL, Adler A, Conklin BR, Hood L, Kuiper M, Sander C, Schmulevich I, Schwikowski B, Warner GJ (2007). Integration of biological networks and gene expression data using Cytoscape. Nat Protocols.

[116] Shannon P, Markiel A, Ozier O, Baliga NS, Wang JT, Ramage D, Amin N, Schwikowski B, Ideker T (2003). Cytoscape: a software environment for integrated models of biomolecular interaction networks. Genome Res.

