# Supplementary material for: A systems biology approach identified different regulatory networks targeted by KSHV miR-K12-11 in B cells and endothelial cells
Source: BMC Genomics. 2014 Aug 8;15(1):668. doi: 10.1186/1471-2164-15-668 (PMC4147158; doi:10.1186/1471-2164-15-668)
Supplement: Supplementary file 2 — Additional file 2: Supplemental tables (Table S1-S3) and figures (Figure S1-S3). (PDF 276 KB) [file 12864_2014_6372_MOESM2_ESM.pdf]

Table S1. Gene sets retrieved from Geo or ArrayExpress using search terms "miR-155" or "miR-K12-11"

list	Accession	Gene #	Description	Reference
**1**	GSE13296	45	regulated genes were identified in LPS-activated moDCs after miR-155 knockdown	[1]
**2**	GSE14477	261	changed genes after overexpress microRNA-155 in lung fibroblasts	[2]
**3**	GSE10467	10	genes regulated by miR-155 in a mouse macrophage cell line	[3]
**4**	GSE10863, GSE10864, GSE10868	78	genes expressed more than twofold lower or twofold higher in miR-155 expressing cells	[4]
**5**	GSE8867	64	genes responding to miR-K12-11 overexpression in BJAB cells	[5]
**6**	GSE9264	66	genes changed by both miR-155 and miR-K12-11 in 293 cells	[6]

Table S2. Algorithms for miRNA target prediction

Algorithm	Criteria for Prediction and Ranking	Website	Reference
TargetScan	Stringent seed pairing, site number, site type, site context (which includes factors that influence site accessibility); option of ranking by likelihood of preferential conservation rather than site context	http://targetscan.org	[7]
EMBL	Stringent seed pairing, site number, overall predicted pairing stability	http://russell.embl-heidelberg.de	[8]
PicTar	Stringent seed pairing for at least one of the sites for the miRNA, site number, overall predicted pairing stability	http://pictar.mdc-berlin.de	[9]
EIMMo	Stringent seed pairing, site number, likelihood of preferential conservation	http://www.mirz.unibas.ch/ElMMo2	[10]
Miranda	Moderately stringent seed pairing, site number, pairing to most of the miRNA	http://www.microrna.org	[11]
miRBase Targets	Moderately stringent seed pairing, site number, overall pairing	http://microrna.sanger.ac.uk	[12]
PITA	Moderately stringent seed pairing, site number, overall predicted pairing stability, predicted site accessibility	http://genie.weizmann.ac.il/pubs/mir07/mir07_data.html	[13]
mirWIP	Moderately stringent seed pairing, site number, overall predicted pairing stability, predicted site accessibility	http://146.189.76.171/query	[14]

RNA22	Moderately stringent seed pairing, matches to sequence patterns generated from miRNA set, overall predicted pairing and predicted pairing stability	http://cbcsrv.watson.ibm.com/rna22.html	[15]
RNAhybrid	thermodynamic stability, Moderately stringent seed pairing	http://bibiserv.techfak.uni-bielefeld.de/rnahybrid/	[16]
Targetboost	Moderately stringent seed pairing; site number, conservation; thermodynamic stability	http://www.interagon.com/demo/	[17]

Table S3 Enrichment pathways and associated genes. Genes in bold are also putative direct targets of miR-K12-11.

**Biological process**	**Genes in TIVE**	**Genes in BJAB**
**IFN-γ signaling pathway**	HILA-A, HLA-B, HLA-C, HLA-DMA, HLA-F, HLA-G, IFI30, IRF1, IRF7, IRF9, OAS1, **OAS2**, OAS3, OASL, **SOCS1**, **STAT1**	CAMK2G, HLA-C, HLA-E, **IFNGR1**, IRF3, IRF9, PTPN6, SP100
**Response to glucose stimulus/ carbohydrate metabolic process**	**AKR7A2**, B4GALT1, CS, GALT, **GBA,** GOT1, GYG1, NPL, NUP160, **NUP43,** **PFKFB2**, PFKFB3, **PGK1,** PRPS1	CTSB, **EP300**, PFKL, RHOC, SREBF1, **TCF7L2**, UCP2

**induction of apoptosis/ Regulation of apoptotic process**	**ACSL5**, APP, ATPIF1, BAD, BCL2A1, BEX2, BIRC5, BTG1,CASP3, **CASP9**, CD70, **CEBPB**, DEDD, **DUSP1**, **ERN1**, FEM1B, **FOXO3**, **GCH1**, HIP1, IRF1, **JMY**, KLF10, MAPK1, **MUL1**, MX1, NACC1, NDUFA13, NDUFS3, NUDT2, PRMT2, PRMT2, **PRDX2**, PSMB2, **PSMB3**, PSMB6, **PSMB8**, PSMB9, PSMC3, PSMD13, PSMD8, PSMD13, PSMG2, **PTEN**, RNF41, **SKI,** SKIL, **STAT1, STK17A**, STK17B, USP7	--

Figure S1 Different components of the same IFN pathway were targeted in TIVE and BJAB cells. Green : unchanged ; Blue: up-regulated; Pink: down-regulated; Pink boxes with red words: down-regulated genes that are potential direct targets. Up: in BJAB cells, the cytokine receptor may be directly targeted by miR-K12-11, leading to

reduced levels of downstream factors. Down: in TIVE cells, the transcription factor STAT and AKT are directly targeted, amplifying the effect to a large set of genes.

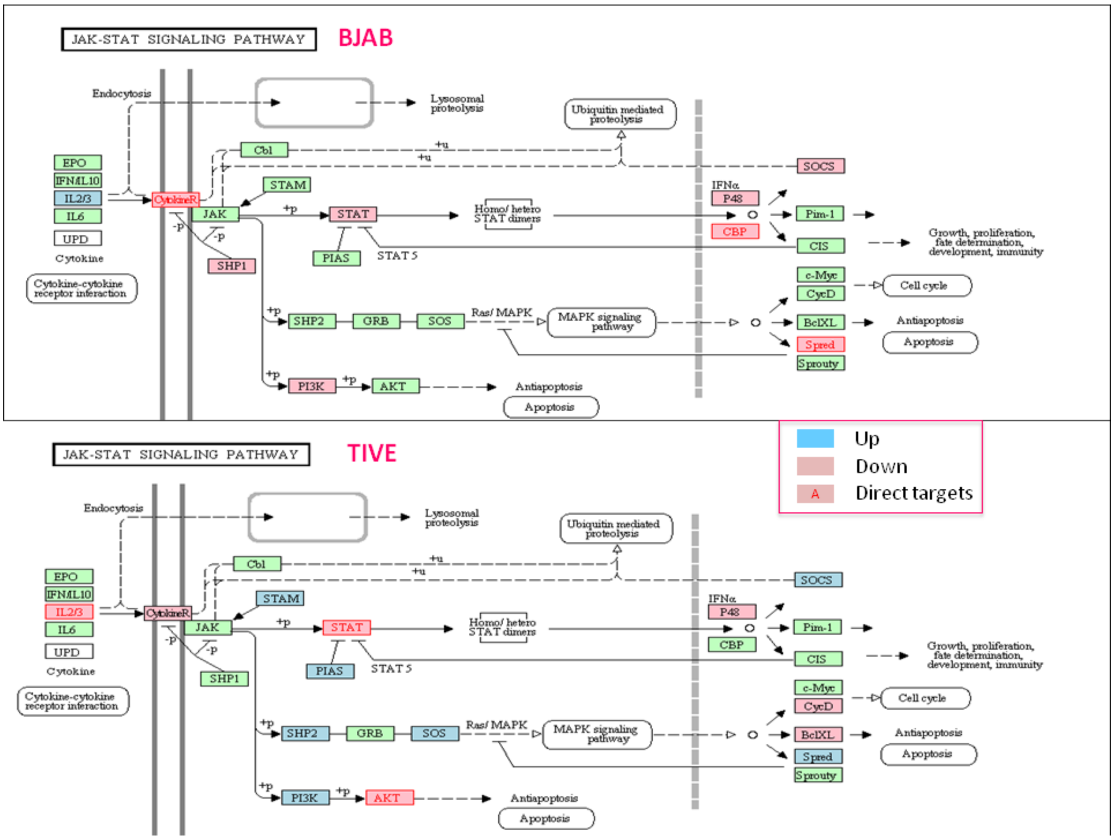

Figure S2 Enrichment of down-regulated genes in the neighboring genes of CASP9 centered network of protein interaction.

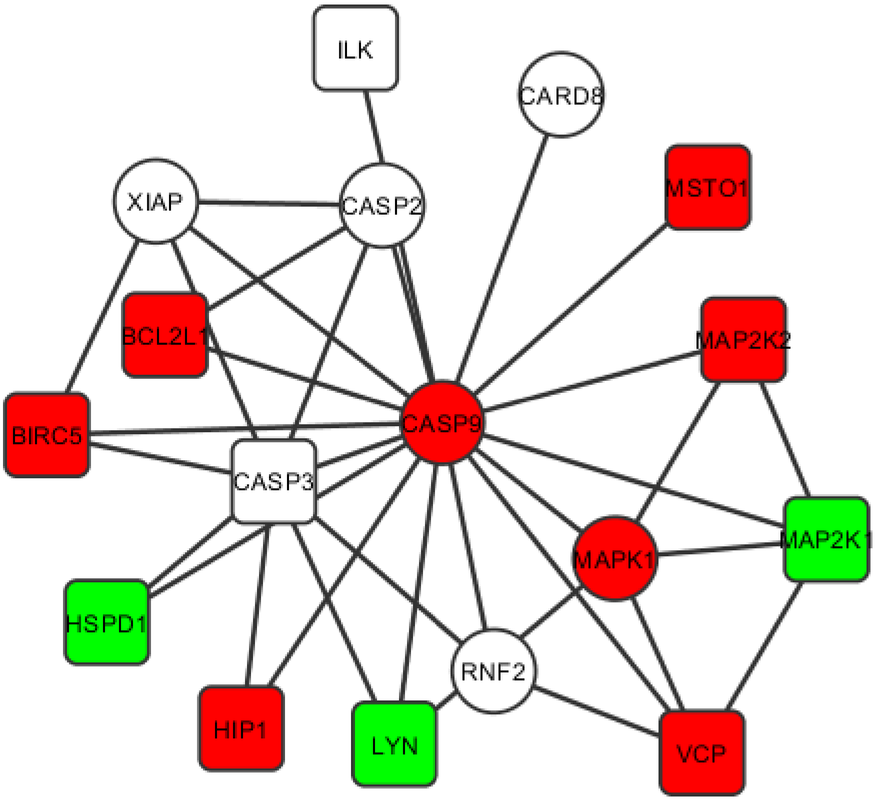

Figure S3 Connectivity of human protein-protein interactions. The distribution follows the power law. Few proteins have many neighbors, while most genes are sparsely connected.

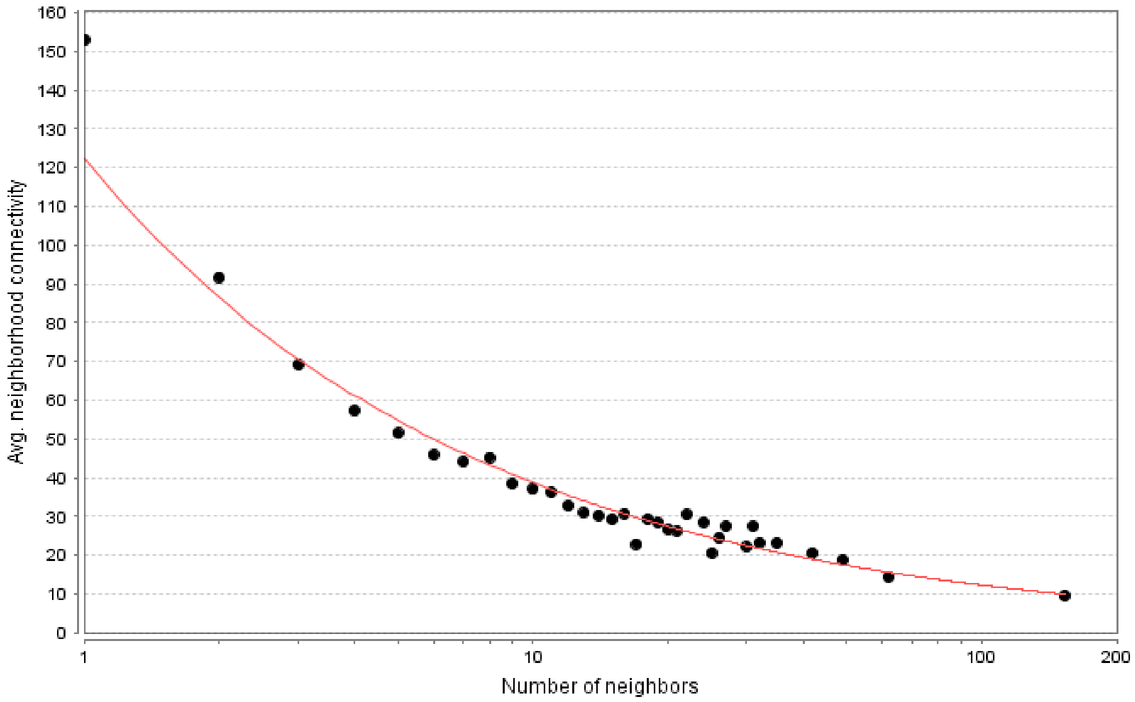

1. Ceppi M, Pereira PM, Dunand-Sauthier I, Barras E, Reith W, Santos MA, Pierre P: **MicroRNA-155 modulates the interleukin-1 signaling pathway in activated human monocyte-derived dendritic cells**. *Proc Natl Acad Sci U S A* 2009, **106**(8):2735-2740.
2. Pottier N, Maurin T, Chevalier B, Puisségur MP, Lebrigand K, Robbe-Sermesant K, Bertero T, Lino Cardenas CL, Courcot E, Rios G *et al*: **Identification of keratinocyte growth factor as a target of microRNA-155 in lung fibroblasts: implication in epithelial-mesenchymal interactions**. *PLoS One* 2009, **4**(8):e6718.
3. O'Connell RM, Rao DS, Chaudhuri AA, Boldin MP, Taganov KD, Nicoll J, Paquette RL, Baltimore D: **Sustained expression of microRNA-155 in hematopoietic stem cells causes a myeloproliferative disorder**. *J Exp Med* 2008, **205**(3):585-594.
4. Yin Q, McBride J, Fewell C, Lacey M, Wang X, Lin Z, Cameron J, Flemington EK: **MicroRNA-155 is an Epstein-Barr virus-induced gene that modulates Epstein-Barr virus-regulated gene expression pathways**. *J Virol* 2008, **82**(11):5295-5306.
5. Gottwein E, Mukherjee N, Sachse C, Frenzel C, Majoros WH, Chi JT, Braich R, Manoharan M, Soutschek J, Ohler U *et al*: **A viral microRNA functions as an orthologue of cellular miR-155**. *Nature* 2007, **450**(7172):1096-1099.
6. Skalsky RL, Samols MA, Plaisance KB, Boss IW, Riva A, Lopez MC, Baker HV, Renne R: **Kaposi's sarcoma-associated herpesvirus encodes an**

ortholog of miR-155. *Skalsky RL, Samols MA, Plaisance KB, Boss IW, Riva A, Lopez MC, Baker HV, Renne R* 2007, **81**(23):12836-12845.

7. Friedman RC, Farh KK, Burge CB, Bartel DP: **Most mammalian mRNAs are conserved targets of microRNAs**. *Genome Res* 2009, **19**(1):92-105.
8. Stark A, Brennecke J, Bushati N, Russell RB, Cohen SM: **Animal MicroRNAs confer robustness to gene expression and have a significant impact on 3'UTR evolution**. *Cell* 2005, **123**(6):1133-1146.
9. Lall S, Grün D, Krek A, Chen K, Wang Y-L, Dewey CN, Sood P, Colombo T, Bray N, MacMenamin P *et al*: **A Genome-Wide Map of Conserved MicroRNA Targets in C. elegans**. *Current Biology*, **16**(5):460-471.
10. Gaidatzis D, van Nimwegen E, Hausser J, Zavolan M: **Inference of miRNA targets using evolutionary conservation and pathway analysis**. *BMC Bioinformatics* 2007, **8**:69.
11. Betel D, Wilson M, Gabon A, Marks DS, Sander C: **The microRNA.org resource: targets and expression**. *Nucleic Acids Res* 2008(36 Database):D149 - D153.
12. Griffiths-Jones S: **The microRNA Registry**. *Nucleic Acids Res* 2004, **32**(Database issue):D109-111.
13. Kertesz M, Iovino N, Unnerstall U, Gaul U, Segal E: **The role of site accessibility in microRNA target recognition**. *Nat Genet* 2007, **39**(10):1278-1284.
14. Hammell M, Long D, Zhang L, Lee A, Carmack CS, Han M, Ding Y, Ambros V: **mirWIP: microRNA target prediction based on microRNA-containing ribonucleoprotein-enriched transcripts**. *Nat Methods* 2008, **5**(9):813-819.
15. Miranda KC, Huynh T, Tay Y, Ang YS, Tam WL, Thomson AM, Lim B, Rigoutsos I: **A pattern-based method for the identification of MicroRNA binding sites and their corresponding heteroduplexes**. *Cell* 2006, **126**(6):1203-1217.
16. Krüger J, Rehmsmeier M: **RNAhybrid: microRNA target prediction easy, fast and flexible**. *Nucleic Acids Res* 2006, **34**(Web Server issue):W451-454.
17. Saetrom O, Snove O, Jr., Saetrom P: **Weighted sequence motifs as an improved seeding step in microRNA target prediction algorithms**. *Rna* 2005, **11**(7):995-1003.
